# Location-dependent effects of trauma on oxidative stress in humans

**DOI:** 10.1371/journal.pone.0205519

**Published:** 2018-10-11

**Authors:** Luis Servia, José C. E. Serrano, Reinald Pamplona, Mariona Badia, Neus Montserrat, Manuel Portero-Otin, Javier Trujillano

**Affiliations:** 1 Department of Critical Care Unit, University Hospital Arnau de Vilanova, University of Lleida-IRBLleida, Lleida, Spain; 2 Department of Experimental Medicine, Faculty of Medicine, University of Lleida-IRBLleida, Lleida, Spain; Hospital Universitari Bellvitge, SPAIN

## Abstract

Though circulating antioxidant capacity in plasma is homeostatically regulated, it is not known whether acute stressors (i.e. trauma) affecting different anatomical locations could have quantitatively different impacts. For this reason, we evaluated the relationship between the anatomical location of trauma and plasma total antioxidant capacity (TAC) in a prospective study, where the anatomical locations of trauma in polytraumatic patients (n = 66) were categorized as primary affecting the brain -traumatic brain injury (TBI)-, thorax, abdomen and pelvis or extremities. We measured the following: plasma TAC by 2 independent methods, the contribution of selected antioxidant molecules (uric acid, bilirubin and albumin) to these values and changes after 1 week of progression. Surprisingly, TBI lowered TAC (919 ± 335 μM Trolox equivalents (TE)) in comparison with other groups (thoracic trauma 1187 ± 270 μM TE; extremities 1025 ± 276 μM TE; p = 0.004). The latter 2 presented higher hypoxia (PaO_2_/FiO_2_ 272 ± 87 mmHg) and hemodynamic instability (inotrope use required in 54.5%) as well. Temporal changes in TAC are also dependent on anatomical location, as thoracic and extremity trauma patients’ TAC values decreased (1187 ± 270 to 1045 ± 263 μM TE; 1025 ± 276 to 918 ± 331 μM TE) after 1 week (p < 0.01), while in TBI these values increased (919 ± 335 to 961 ± 465 μM TE). Our results show that the response of plasma antioxidant capacity in trauma patients is strongly dependent on time after trauma and location, with TBI failing to induce such a response.

## Introduction

It is generally assumed that critical care patients, as well as experimental models of acute trauma, show an increase in tissue oxidative stress (OS), as indicated by systematic reviews [1]. This may be the basis for the positive response to some antioxidant treatments in this context [2]. In this clinical setting, OS is derived from a concerted build-up of proinflammatory factors, leading to increased tissue efflux of reactive species (RS), such as oxygen-, nitrogen- and carbonyl species [1]. Physiologically, the deleterious effects of RS are limited by antioxidant defense systems, which counteract their potential to modify biomolecules. This modification potential is based on the ability of RS to modify biomolecules, both structurally and functionally, exemplified by their capacity for molecular disruption of lipids, proteins and nucleic acids. For these reasons, oxidative stress has been invoked in multiple organ failure (MOF) after systemic inflammatory response syndrome (SIRS) following traumatic events [3,4].

Though there are many techniques to evaluate oxidative stress status, a consensus on which is the best measure does not exist [5–7]. This may be due to the diverse nature of RS and the myriad of potential targets. However, clinically applicable methods should include general measurements that do not require specialized equipment. Specifically, one could try to analyse RS directly, but its low stability does not allow for precise quantification in the clinical environment. On the other hand, one could measure the molecular footprints generated by RS on selected biomolecules, the so-called OS biomarkers, but specific detection requires forefront mass spectrometry and dedicated chromatographic systems. In contrast, the measurement of antioxidant molecules determined in the plasma compartment by the total antioxidant capacity (TAC) technique has been used as a robust oxidative stress indicator [7,8], assuming that, in the event of continuous OS, TAC may be exhausted. Plasma TAC is known as the result of the combination of several biomolecules, such as classical antioxidant vitamins (C and E), sulfhydryl groups from plasma proteins, as well as some degradation products such as bilirubin and uric acid, besides other uncharacterized antioxidants [9].

Accounting for this diversity, most previous studies aiming to evaluate oxidative status in the critical care setting have been focused on septic shock or sepsis with SIRS and/or MOF. These studies have shown an increase in RS-derived markers concurrently with decreases in the antioxidant levels in plasma, with those patients with a more critical status showing a higher degree of oxidative modification [10].

Although these findings indicate an influence of clinical status in OS homeostasis based on patients with sepsis, relatively fewer reports have focused on trauma patients [11, 12]. Disease severity is apparently similar in sepsis and trauma patients, but their clinical specificities could help to delineate the role of RS in the critical care patient outcome. As an example of these specificities, generally, traumatic patients are considered as a good example of abrupt changes in homeostasis, while sepsis patients often have a longer prodromic phase. Furthermore, trauma patients are often younger and without major comorbidities. Reinforcing the specificity of the relationship between the severity of clinical status and OS, we demonstrated that plasma TAC values were strongly influenced by trauma severity (APACHE II) and its gross location, with a paradoxical (in comparison to sepsis patients) increase in TAC values with increasing severity [13]. The aim of this study was to obtain a more accurate estimation of the potential influence of the anatomical location of trauma on blood antioxidant status. In the present work, we have selected those patients with a unique lesion located almost exclusively in a single, defined anatomic place. In addition, to evaluate the influence location in OS-related parameters in plasma, we focused to the individual changes in these after a 7-day period in an intensive care unit (ICU) environment.

## Materials and methods

### Study design, patient population and reagents

A prospective cohort study was performed in the ICU of the Arnau de Vilanova University Hospital (Lleida, Spain). The protocol was approved by the Institutional Ethics Committee of the Arnau de Vilanova Hospital. All participants (or their legal representatives) gave their written consent for the study. As a healthy population, we included plasma samples from fasted individuals (n = 49, 24 females) with a mean age not different from that of trauma-affected individuals. The healthy population was used to establish the reference values of ferric reducing activity/antioxidant power (FRAP) and 2,2'-azino-bis(3-ethylbenzothiazoline-6-sulphonic acid (ABTS). For the rest of the biomarkers, the reference ranges established by our laboratory were used.

The inclusion criteria for trauma patients were all trauma-affected patients with severe status requiring ICU admission caused by a traumatic event in the prior 48 h, age >16 years, with a follow-up period of 7 days in the ICU, in the period between 2011/10/1 and 2012/11/15.

Patient acuity was defined according to the Injury Severity Score (ISS) [14]. Anatomical influence was evaluated according to the Abbreviated Injury Scale (AIS) [15]. Anatomical locations of trauma were defined according to the following scheme: TBI (traumatic brain injury), ABD (abdominal trauma), THORAX (thoracic trauma) and PEXT (pelvis or extremity trauma). Severity of Injury is ranked on a scale of 1–6 as follows: (1 = minor, 2 = moderate, 3 = serious, 4 = severe, 5 = critical and 6 = maximum) according to the AIS classification [15]. In the present work, we only include those patients with a major single trauma (TBI, ABD, THORAX or PEXT), with an AIS ≥3 without any other location with AIS ≥2, according previously published criteria [16].

The exclusion criteria were all patients whose samples were not obtained in the first 48 h, those discharged before 7 days and all chronic disease patients (according APACHE II) [17]. Similarly, we excluded patients with acute kidney failure, defined as plasma creatinine ≥1.5 mg/dl or diuresis <400 ml/24 h.

Age, gender, length of ICU stay, APACHE II [17] classification and mortality were also documented. We also recorded the severity of loss of respiratory function, measured as the lowest PaO_2_/FiO_2_ in the first 24 h of ICU admission, by using arterial gasometry. Hemodynamic instability (shock) was diagnosed as the patient having a maintained (>2 h) low systolic blood pressure level (<90 mm Hg) and/or requiring norepinephrine. The Glasgow Coma Scale (GCS) was used for evaluation of neurological function. The TBI group was divided into moderate (GCS 9–12 points) and severe (Glasgow <9) involvement. Transfusion need was defined as those patients requiring more than one hemoconcentrate, and coagulopathy was defined as Quick score <70% and/or thrombocyte count <100,000/μL. All scales were validated by 2 independent evaluators.

### Sample preparation and total antioxidant capacity (TAC) measurement

Measurements were performed in a double-blinded fashion; therefore, samples were randomized before TAC analyses. Blood was obtained in all cases by standard venipuncture or central venous catheterisation (basal measurement: between 24 and 48 h after the traumatic event and 7 days later in sodium citrate tubes. Immediately after sampling, diethylpentaacetic acid (1 mM) and butyl-hydroxy-toluene (10 μM) were added to the sample, in order to avoid artefactual oxidation. Plasma was obtained after centrifugation (2500 rpm, 4 ºC, 10 min), and aliquoted into cryovials for immediate storage at −80°C.

TAC was measured using 2 methods: FRAP and the capacity for neutralization of the free radical ABTS, which measures the reducing ability and the proton transfer capacity, respectively. For the FRAP assay, 900 μL of the FRAP reagent containing 2,4,6-tri(2-piridil)-s-triazine, FeCl_3_ and acetate buffer (300 mM, pH 3.6) were mixed with 90 μL of distilled water and 30 μL of plasma or sodium phosphate buffer for the blank. Maximum absorbance values of this solution at 595 nm were taken at 37°C, using a Beckman DU-640 spectrophotometer (Beckman Instruments Inc., Fullerton, CA, USA). Antioxidant capacity was referenced to standards containing known concentrations of 6-hydroxy-2,5,7,8- tetramethylchroman-2-carboxylic acid (Trolox) and expressed as μM Trolox Equivalents (TE) [18].

For the ABTS assay, ABTS radical cation (ABTS^+^·) was produced by reacting 7 mmol/L ABTS stock solution with 2.45 mmol/L potassium persulphate in the dark at room temperature for 12–16 h before use. The ABTS^+^· solution was diluted with 5 mM phosphate buffered saline (pH 7.4) to an absorbance of 0.70±0.02 at 730 nm. After addition of 0.1 mL of plasma to 3.9 mL of diluted ABTS^+^· solution, absorbance readings were taken every 20 s using a Beckman DU-640 spectrophotometer. The reaction was monitored for 6 min. Inhibition of absorbance vs. time was plotted, and the area below the curve (0–6 min) was calculated. Solutions of known Trolox concentration were used to calculate antioxidant capacity equivalents [18]. Inter-assay and intra-assay coefficients of variance were below 5%.

To evaluate the contribution of structurally characterized plasma antioxidants, the concentrations of uric acid (reference range (RR): 3.5–7.2 mg/dL), bilirubin (RR 0.30–1.30 mg/dL), protein (RR 6.0–8.3 g/dL) and albumin (RR 3.4–5.2 g/dL) were also measured by routine biochemical analysis in a Cobas autoanalyzer at the Clinical Laboratory of Arnau Vilanova University hospital.

### Statistical methods

The data are presented either as the mean ± standard deviation, the median (interquartile range) or as a percentage. For betwee-group comparisons, the chi-squared (χ^2^) test was used for categorical variables, and the Kruskal-Wallis test or Wilcoxon test were used for continuous variables. The relationship between the TAC values and markers was assessed by Spearman’s correlation test. The calculations were performed using SPSS software version 20.0. For Circos table representation, we employed the package reported in Krzywinski et al. [19].

## Results

### Clinical characteristics

A total of 66 participants were enrolled in this study, and their general characteristics are shown in Table 1. As expected, TBI patients showed lower GCS test scores, THORAX patients had lower (PaO_2_/FiO_2_) respiratory function and those with PEXT had a higher shock prevalence and greater transfusion needs.

**Table 1:** Study population characteristics of trauma patients (according anatomic location).

		ANATOMIC AREA				
	ALL (n = 66)	TBI (n = 20)	ABD (n = 8)	THORAX (n = 27)	PEXT (n = 11)	*p*
**Age** (years)^a^	47±22	47±22	46±17	53±15	36±18	*0*.*085*
**Gender** male	78.8	75.0	87.5	88.9	54.5	*0*.*109*
**APACHE I**I^a^	12±6	13±5	12±7	10±6	11±8	*0*.*141*
**ISS**^a^	19±8	21±10	20±9	17±8	18±7	*0*.*713*
**Type of trauma**						
Transit	62.1	55.0	75.0	59.3	72.7	*0*.*489*
Work-related	7.6	5.0	0.0	7.4	18.2	
Other	30.3	40.0	25.0	33.3	9.1	
**MV**	43.9	60.0	75.0	18.5	54.5	*0*.*005*
**PaO2/FiO2**	318±110	361±113	308±131	272±87	354±102	*0*.*040*
**Shock-NA**	31.8	30.0	37.5	22.2	54.5	*0*.*271*
**Glasgow** (score)	15 (12–15)	11 (5–12)	13 (9–15)	15 (15–15)	15 (14–15)	*0*.*001*
**Blood transfusion**^b^	21.2	10.0	12.5	14.8	63.6	*0*.*002*
**ICU stay (days)**^a^	21±24	22±20	32±39	19±25	15±8	*0*.*831*
**ICU Mortality**	7.6	10.0	12.5	3.7	9.1	*0*.*787*

ISS (Injury severity score), FiPO_2_ (Inspirational Fraction of O_2_), MV: mechanical ventilation. Values are shown as % unless stated otherwise.

^a^ mean ± SD.

^b^Median (IQR). Blood transfusion (considering a minimum amount of 1 haemoconcentrate required).

*P* values shown are according χ^2^ or Kruskal-Wallis test.

The extraction was performed by standard venipuncture in only 5 patients and in all remaining cases, by central venous catheter. We did not find any differences in clinical or analytical values.

### Plasma TAC values

Compared with healthy individuals, the values of TAC measured by FRAP in trauma patients were higher, while those of ABTS were lower (95% CI of the mean: 463–501 and 2130–2216 for FRAP and ABTS in a healthy population, respectively). In all anatomic locations, there was a reduction in FRAP and an increase in ABTS values at day 7 after ICU entry, the increase in ABTS values being significant in all trauma locations and larger in PEXT trauma. In general terms, it was also observed that TAC values measured by both methods approached the values found in the healthy population as the number of days spent in the ICU increased. As shown in Table 2, at ICU entry, FRAP values were significantly lower in TBI and ABD in comparison with THORAX and PEXT patients (p = 0.014). However, after 1 week, these values tended to increase in TBI and ABD patients, whereas THORAX and PEXT patients showed a decrease, leading to lack of significant differences between locations (p = 0.419). ABTS measurements showed a tendency (p = 0.055) toward lower values in TBI and PEXT, with a tendency to increase in all cases after 1 week, being significant in the case of PEXT patients (p < 0.05).

**Table 2:** Values of TAC and its components according to anatomic location and time after ICU entry.

			ANATOMIC AREA				
	Control	ALL (n = 66)	TBI (n = 20)	ABD (n = 8)	THORAX (n = 27)	PEXT (n = 11)	*p*^*1*^
TAC							
**FRAP** (μM TE)	1	1043 ± 312	919 ± 335	915 ± 283	1187 ± 270	1025 ± 276	*0*.*014*
	7	987 ± 348	961 ± 465	948 ± 324	1045 ± 263	918 ± 331	*0*.*419*
**ABTS** (μM TE)	1	1661 ± 520	1502 ± 598	1958 ± 280	1778 ± 462	1459 ± 509	*0*.*055*
	7	1732 ± 556*	1572 ± 634	1976 ± 266	1803 ± 554	1697 ± 534*	*0*.*325*
MARKERS							
**Uric acid** (mg/dL)	1	3.53 ± 3.5	1.99 ± 1.3	3.00 ± 2.2	4.74 ± 5.2	3.83 ± 2.2	*0*.*007*
	**7**	3.11 ± 4.0*	1.71 ± 1.4	2.73 ± 1.9*	3.65 ± 4.0*	4.58 ± 7.1	*0*.*048*
**Bilirubin** (mg/dL)	1	0.99 ± 0.6	0.99 ± 0.6	0.61 ± 0.3	0.85 ± 0.7	0.99 ± 0.8	*0*.*283*
	**7**	0.98 ± 1.6*	0.74 ± 0.5*	0.59 ± 0.3	1.21 ± 2.5	1.15 ± 1.1	*0*.*852*
**Total protein** (mg/dL)	1	4.92 ± 0.8	5.21 ± 0.9	4.99 ± 0.9	5.00 ± 0.6	4.16 ± 0.6	*0*.*008*
	**7**	5.51 ± 0.7*	5.55 ± 0.7*	5.46 ± 0.8*	5.64 ± 0.7*	5.18 ± 0.6*	*0*.*435*
**Albumin** (mg/dL)	1	3.16 ± 0.5	3.31 ± 0.5	3.27 ± 0.6	3.23 ± 0.5	2.65 ± 0.5	*0*.*012*
	7	3.17 ± 0.6	3.21 ± 0.6	3.25 ± 0.7	3.27 ± 0.6	2.88 ± 0.6	*0*.*375*

Values are shown as means ± SD. ***p***^***1***^ (shows *P* values according to the Kruskal-Wallis test measuring differences between anatomic locations. Control 1: first 2 days; Control 7: 1 week.

* indicates significant differences between control 1 and 7 according to the Wilcoxon test (p < 0.05).

TE: Trolox-equivalents.

### Relationship of TAC values with individual molecules

As indicated in Table 2, uric acid concentrations were increased in the THORAX group relative to the TBI and ABD groups (p < 0.007). After 1 week, these values decreased, with the exception of PEXT patients. In line with this behaviour, bilirubin values were highest in the TBI and ABD groups. In contrast with uric acid, bilirubin values decreased in TBI and ABD, while they increased in THORAX and PEXT. Finally, both total proteins and serum albumin showed the same pattern, increasing in the week after ICU entry, with the lowest values in the PEXT group at entry (p < 0.008).

We also analysed the TBI group separately according to the degree of affectation (moderate vs severe). We did not find differences in the demographic variables. However, there were statistically significant differences between the severe and moderate groups in APACHE II (23 ± 11 vs 18 ± 10, p = 0.002) and MV need (83.3 vs 25.0%, p = 0.009). The moderate group did not present mortality, and there were 2 deaths in the severe group (not statistically significant; p = 0.224). Regarding the values of TAC and its components, we only found differences in the levels of FRAP in the first control (786 ± 270 vs 928 ± 535 uM TE, p = 0.025).

Treating their behaviour as continuous variables, we found a significant correlation between the 2 TAC measurements, as well as between uric acid values and TAC measurements (Table 3). Bilirubin values were inversely related to TAC. APACHE II scores were inversely correlated with FRAP.

**Table 3:** TAC correlation with clinical variables.

	ALL (n = 66)							
	FRAP	ABTS	Uric Acid	Bilirubin	Protein	Albumin	APACHE II	Glasgow
**FRAP** (μM TE)	—-							
**ABTS** (μM TE)	**0.360**	—-						
**Uric acid** (mg/dL)	**0.588**	0.128	—-					
**Bilirubin** (mg/dL)	- 0.166	**- 0.333**	- 0.213	—-				
**Protein** (mg/dL)	0.140	0.216	0.122	- 0.048	—-			
**Albumin** (mg/dL)	0.105	0.176	0.091	- 0.012	**0.892**	—-		
**APACHE II**(score)	**- 0.251**	0.038	**- 0.229**	0.198	**- 0.273**	**- 0.324**	—-	
**Glasgow** (score)	**0.458**	0.057	**0.479**	- 0.129	- 0.094	- 0.130	**- 0.556**	—-
**PaO**_**2**_**/FiO**_**2**_	- 0.134	- 0.197	- 0.164	0.159	0.137	0.082	- 0.156	- 0.197

Correlations according Spearman’s rank correlation test. Values shown are those present in the first 48 h. Significant correlations are shown in bold characters (p < 0.05). TE: Trolox-equivalents

**Fig 1:**
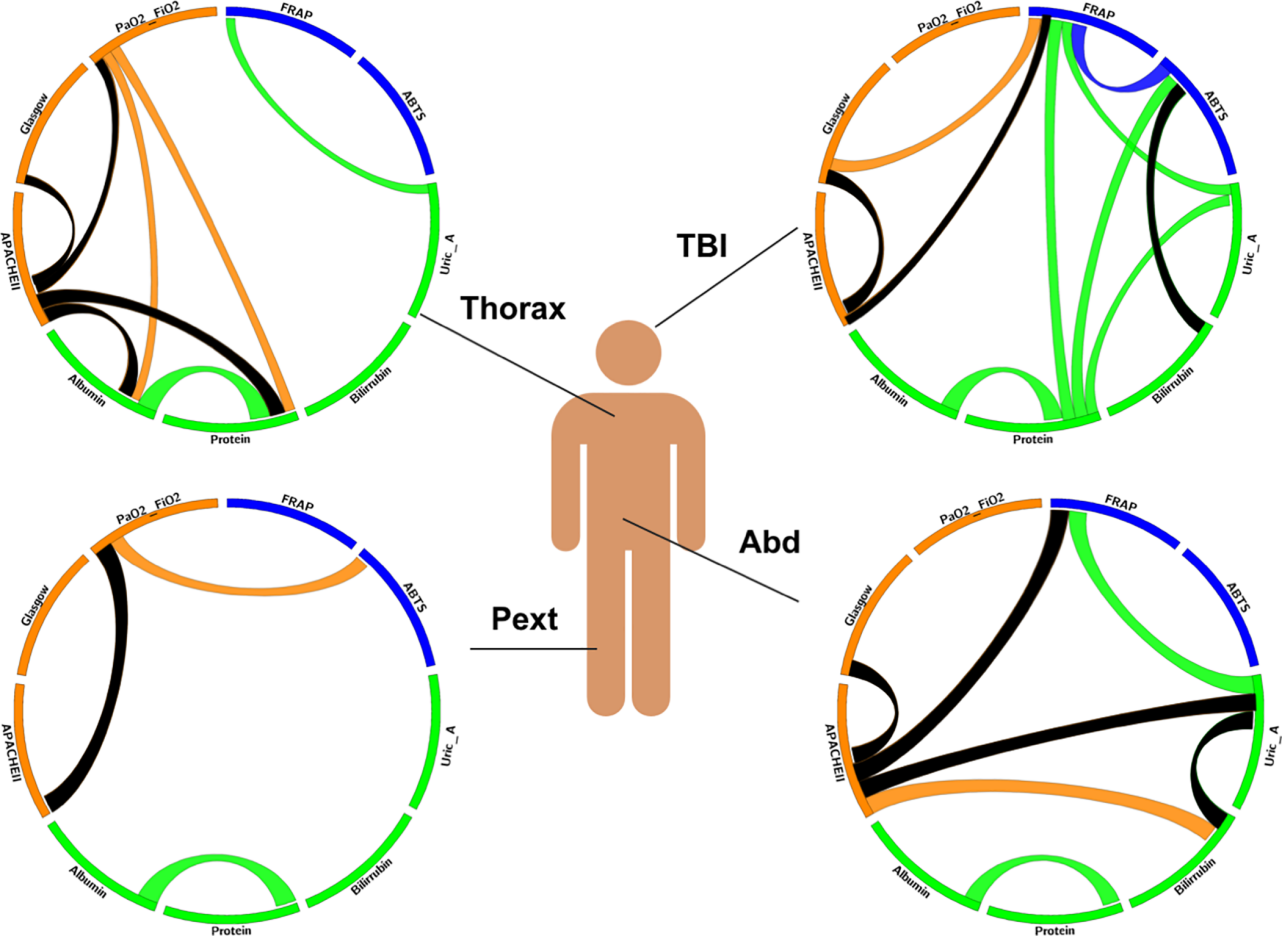
Cord diagrams showing that correlations between clinical, analytical and plasma antioxidant variables are dependent on trauma location. Individual (one for each location) Circos-type graphs show the degree of correlation, where circular segments denotate the variables evaluated and lines connecting them, the existence of significant correlations according to Spearman’s rank correlation test (p < 0.05); negative correlations are marked in black. Values used are those present in the first 48 h.

Reinforcing the influences of anatomic location on the relationship between TAC and severity, we demonstrated a difference in these relationships between the analysed groups (Fig 1). Thus, ABD patients maintained the correlation of FRAP with uric acid but not with ABTS. Moreover, they showed an inverse correlation between APACHE II score and bilirubin values. Similarly, THORAX patients did not maintain the correlation between the 2 measurements of TAC. Finally, PEXT patients showed a significant correlation between PaO_2_/FiO_2_ and ABTS.

## Discussion

The measurement of TAC is not a common feature in clinical diagnosis, mainly because of the lack of standardization of the methodology employed and the need for standardization of the normal values and range found in the normal population. Trauma patients and healthy individuals presented different levels, though ABTS and FRAP values showed inverse changes [13]. Previous data in ICU patients showed similar results: while some patients with SIRS or sepsis show decreased ABTS values, septic shock patients show an increase in this variable [20]. In line with our results, other authors evaluating trauma patients have shown increased reductive potential for patients who had experienced severe trauma [12]. Our data demonstrating higher FRAP, but not ABTS, capacity underscores the importance of technique used for determination of antioxidant capacity. Generally, it is assumed that the FRAP assay is more suitable for hydrophilic compounds, in contrast to the ABTS assay, which is suitable for lipophilic compounds. In previous experiments, both sepsis and infusion of lipopolysaccharide increased FRAP capacity [21,22].

It should be remarked that plasma TAC should be the result of the cumulative effects of all the antioxidant compounds (uric acid, bilirubin, albumin, thiols, antioxidant vitamins, exogenous antioxidants) found in the vascular compartment. Although a good correlation was observed between FRAP levels and trauma severity measured by APACHE II and Glasgow scores, no single antioxidant compound was found to correlate with trauma severity scores. Thus, changes in TAC levels could be viewed as an integrative response with global relevance, whereas individual parameters could help to delineate the pathophysiological mechanism [23,24].

Interestingly, our work demonstrates a previously unreported anatomic influence over plasma TAC, which may be explained by pathophysiological mechanisms. Thus, tissue hypoxia (as evidenced by low PaO_2_/FiO_2_ in the THORAX group or by low perfusion in PEXT patients) could help to explain increased uric acid concentrations in these patients, subsequent to ATP degradation. This is shared by sepsis patients, where an increase in uric acid is present [20,25–26], leading to increased TAC values, together with increased bilirubin concentrations. This would arise as a secondary effect from cholestasis induced by sepsis and its treatment [27]. Nonetheless, trauma patients exhibit a high level of haemolysis, secondary to trauma. Similarly, concerning the other component influencing TAC, albumin, it has been reported that hypoalbuminemia in trauma patients can be secondary to low synthetic rates, hypercatabolism or vascular efflux, all leading to diminished TAC [23,28]. In our PEXT group, haemodilution secondary to fluid and haemoderivate treatment could contribute as well to hypoalbuminemia and diminished TAC values in comparison with THORAX patients.

To our surprise, TBI patients showed lower TAC values. Preclinical experimental works show a delayed (24-h post-trauma) RS efflux, with consumption of circulating antioxidants, such as ascorbic acid and tocopherol [29]. We also found a relationship according to the degree of severity of the TBI: patients with more severe TBI had, at an early stage, lower levels of TAC (measured by FRAP) than patients with moderate TBI. These results agree with those obtained in patients with severe head injury, were lipid peroxidation and superoxide dismutase activities were increased, with a concomitant depletion of tocopherol, all related to a poor prognosis [30].

Concerning abdominal trauma, there are few reports on the specific relationship between abdominal location and OS. These previous works conclude that there are no relationships between trauma severity and lipid peroxidation or antioxidant levels [31]. Nonetheless, other works indicate that abdominal trauma, as present in open surgery, induces increased OS [32].

Reinforcing the dissimilarities between trauma and sepsis patients, temporal evolution of TAC values also differs between these groups. Thus, sepsis is associated with a sharp increase in TAC values, followed by a decrease in the subsequent days [20,25]. In our TBI patients, values increased from baseline to 1 week after trauma. In a population with ICU patients with overall SIRS, it was reported that decreased TAC values were a positive prognostic sign [33].

Glutamine and antioxidant-enriched diets are indicated during ICU stay, as a potential measure of inflammation and OS modulation, subsequent to increased free-radical efflux and endogenous antioxidant exhaustion [10,34–35]. However, recent data cast doubts over the clinical effectivity of those dietary measures [36–38], with some works even doubting the nutritional approach as therapeutic support to counter OS [39]. Along these lines, as many as 39% of trauma patients under glutamine supplementation (0.5 g/kg/day) showed decreased glutamine values after 6 days [40]. Nonetheless, our results help to open the question of whether the general use of antioxidant supplements might be effective in these patients, irrespective of trauma location.

The present work is limited by the relatively small population studied as well as the inclusion of patients from a single ICU. Similarly, the non-equal size of the study groups could explain the observed differences, in part on the basis of their high heterogeneity. We also assume that particular dietary habits, such as heavy vitamin/mineral supplementation (before a traumatic event) could influence the starting (and statistically processed) values, especially in small subgroups of subjects (e.g. ABD or PEXT). Furthermore, although the therapeutic approaches chosen for each of the anatomic location groups did not differ significantly, we could not exclude that individually tailored therapeutic approaches may have contributed to the observed differences. Other biomarkers, such as vitamin C or E, with antioxidant capacity that could have modified some of our conclusions could also have been used. Nonetheless, even with these limitations, collectively, our results indicate the complexity of factors affecting circulating antioxidant status in the ICU setting in the context of severe trauma patients. The relationship between antioxidant status and anatomic location as well as the differential results depending on technique used for antioxidant capacity evaluation underscore the need for further studies. Injury can cause oxidative stress and an increase in reactive oxygen species, both of which are mitigated with time. Our main conclusion is that any study carried out on the antioxidant capacity of trauma patients should take into account not only the time since the trauma but also the location of it. Furthermore, these results could be useful in the definition of criteria for inclusion of antioxidant-rich supplements.

In summary, the response of plasma antioxidant capacity in trauma patients, viewed as a homeostatic response, is strongly dependent on time after trauma and location, with TBI failing to induce such a response.
